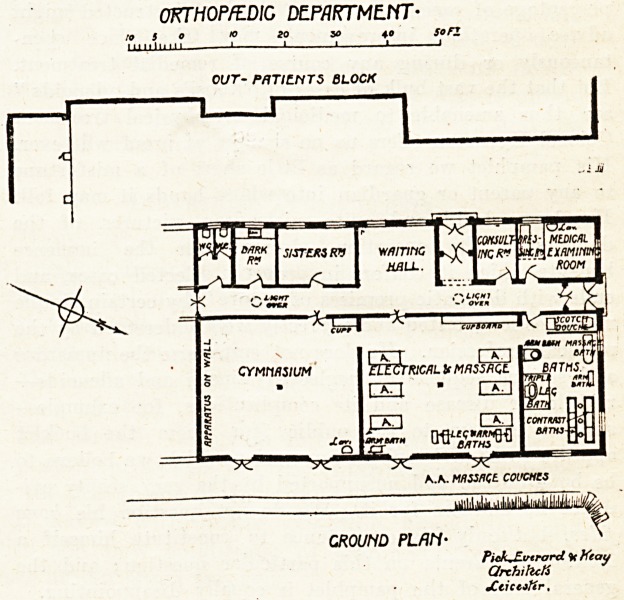# The New Pathological and Orthopædic Blocks

**Published:** 1920-11-20

**Authors:** 


### LEICESTER ROYAL INFIRMARY.

### The New Pathological and Orthopaedic Blocks.

We have received from Messrs. Pick, Everard & Keay, architects, of Leicester, some particulars of the additions to the Leicester Royal Infirmary, of which we are printing certain plans. Two distinct blocks are here illustrated—an Orthopædic Department, measuring 77 feet by 47 feet, and a Pathological Block, which on the ground floor is about 90 feet long. It is 33 feet wide, and the upper story is diminished in length by reason of the fact that nothing is built over the chemical laboratory and the P.M. room, which form the extreme north and south features of the building.

The former block is a fairly complete little installation, the more important elements of which are a good-sized gymnasium and a large general room for electro-therapeutic and massage treatments. Adjoining the latter is a place for baths of various descriptions, and the remainder consists of small apartments—consulting-room, medical examination room, sister's room, etc.—in the centre of which comes the entrance, with suitable waiting-room. The whole block is in contiguity to the general out-patients' block, though not apparently attached thereto.

The larger work—the Pathological Block—was the subject of a ceremony in July last, when the Mayor of Leicester, Mr. Alderman J. Chaplin, declared the building open. Its size has not been determined by the present capacity of the hospital —viz. 300 beds—but the possibility of further future needs has been kept in view. The style of the construction is, we understand, quite simple—local red bricks with concrete sills and copings—and the building is placed at the south-west angle of the hospital grounds. Though only ground and first floors are here illustrated, there is a basement giving space for storage and providing access to the heating and hot-water ducts which communicate presumably with the general heating plant of the main buildings.

Except for the absence of a small museum room, which is often found to be a useful adjunct in a hospital of this size, the design conforms well to the recognised modern requirements of such a pathological department. The mortuary has a refrigerating plant—method not stated—and, except that it is separated by the main passage from the viewing chapel, it appears to be well situated.

We are pleased to recognise that the viewing chapel itself has been designed with due consideration for the reverential atmosphere that such a place ought always to maintain. The waiting-room for visitors is placed close to the entrance, so that friends who come there on their melancholy errand are not in any way introduced to the necessary but disturbing functions of the rest of the building. The P.M. room is well lighted by an ample skylight and has good cross-ventilation by windows. A chemical laboratory (with dark room), a private room (of laboratory character), the sanitary accommodation, and the staircase hall complete the plan of the ground floor.

Upstairs—and the stairs are easy and convenient —the main feature is a large top-lighted and window-lighted bacteriological laboratory, measuring 35 feet by 19 feet. Adjoining it is a histology room, and opening out of the former is a long sterilising room. A good point in this plan is the

**Leicester Royal Infirmary**—(*continued*).

easy access at the head of the stairs to the large asphalte flat over the chemical laboratory. This flat, instead of having the usual low parapets, is surrounded by high walls, thus providing a convenient place for certain work which, though better done out of doors than indoors, is better screened than exposed.

Fireproof construction, tiled walls, and terrazzo floors prevail throughout.

The total cost of this block, including furniture and fittings, was about £10,250. The Orthopædic Block cost £9,100, and when it was opened by the High Sheriff of the County—John Turner, Esq.—on the same day as the Pathological Block it was, largely owing to the gift of £5,000 by the Freemasons of Leicester, free of debt. The governors of the infirmary were also liberal contributors.

The Pathological Block is very appropriately dedicated to the memory of the late Thomas Arnold Johnston, M.D., who died in 1918, and who was honorary physician and first pathologist to the infirmary.

Mr. V. C. Deacon acted as clerk of works in respect of both buildings.

Mr. Feilding Johnson, Jnr., the present Chairman of the Governors, comes of a family which has a long interest in this useful institution. No fewer than four generations of his family were present at the opening ceremony.

## Figures and Tables

**Figure f1:**
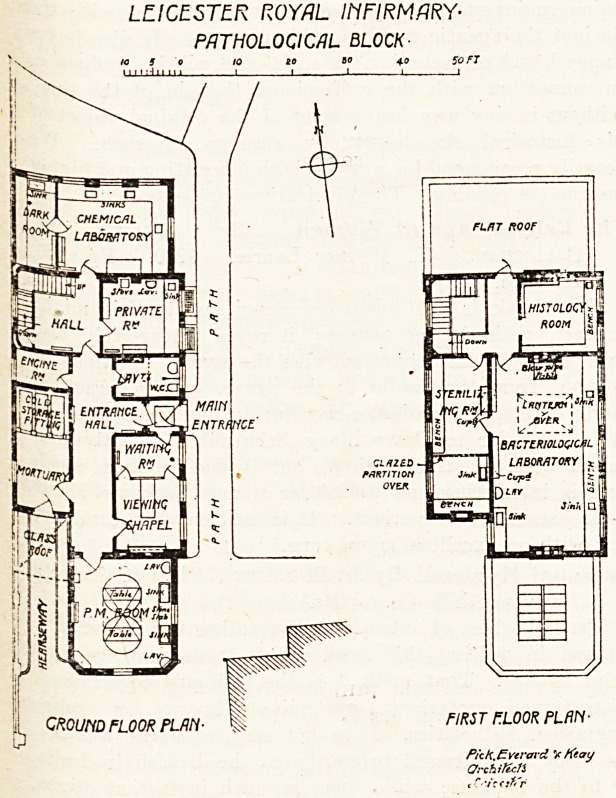


**Figure f2:**